# Efficacy of shock wave therapy and ultrasound therapy in non-insertional Achilles tendinopathy: a randomised clinical trial

**DOI:** 10.3389/fneur.2024.1434983

**Published:** 2024-07-10

**Authors:** Magdalena Stania, Kajetan J. Słomka, Grzegorz Juras, Tomasz Król, Piotr Król

**Affiliations:** ^1^Institute of Sport Sciences, Academy of Physical Education, Katowice, Poland; ^2^Department of Kinesitherapy and Special Methods, School of Health Sciences in Katowice, Medical University of Silesia, Katowice, Poland

**Keywords:** non-insertional Achilles tendinopathy, extracorporeal shock wave therapy, ultrasonic waves, gait analysis, therapeutics

## Abstract

**Objective:**

Physiotherapists and physicians continue to seek effective conservative treatments for Achilles tendinopathy. This study aimed to subjectively and objectively determine the therapeutic efficacy of radial shock wave therapy (RSWT) and ultrasound therapy in non-insertional Achilles tendinopathy.

**Materials and methods:**

Thirty-nine patients with non-insertional Achilles tendinopathy were randomly assigned to three experimental groups, i.e., RSWT (group A), ultrasound therapy (group B), and placebo ultrasound (group C) groups. Before the intervention and at weeks 1 and 6 after the treatment, the patients were assessed using the Victorian Institute of Sport Assessment–Achilles (VISA-A) questionnaire and posturographic measurements of step initiation performed on the force platforms under two different conditions (non-perturbed transit and perturbed transit).

**Results:**

Six weeks after therapy, all groups exhibited significantly increased VISA-A scores against the measurement at week 1 after therapy. The post-therapy percentage changes in VISA-A scores were significantly greater in group A compared to group B. The three-way ANOVA demonstrated that treatment type affected sway range in the frontal plane and mean velocity of the centre of foot pressure displacements in the sagittal and frontal planes during quiet standing before step initiation. The *Bonferroni* post-hoc test showed that the means of all those variables were significantly smaller for group A than for group B patients. The three-way ANOVA revealed an effect of the platform arrangement on transit time and double-support period. The *Bonferroni* post-hoc test revealed statistically longer transit time for the perturbed vs. non-perturbed trials; a reverse relationship was observed for the double-support period.

**Conclusion:**

The VISA-A showed that RSWT was significantly more effective than sonotherapy for alleviation of pain intensity as well as function and activity improvement in patients with non-insertional Achilles tendinopathy. Therefore, RSWT therapy can be used in clinical practice by physiotherapists to alleviate the symptoms of non-insertional Achilles tendinopathy. Objective data registered by force platforms during quiet standing before and after step initiation did not prove useful for monitoring the progress of treatment applied to patients with non-insertional Achilles tendinopathy between consecutive therapy interventions.

**Clinical trial registration:**https://anzctr.org.au/Trial/Registration/TrialReview.aspx?ACTRN=12617000860369, identifier (ACTRN12617000860369).

## Introduction

Achilles tendinopathy results from excessive muscle and/or tendon loading injury and multiple microinjuries (1). Chronic clinical manifestations of Achilles tendinopathy include pain and swelling in the tendon, functional deterioration (2), and stiffness associated with prolonged rest (3). Considering the anatomical criterion, Achilles tendinopathy is classified as midportion tendon dysfunction (non-insertional Achilles tendinopathy), when symptoms are located from 2 to 7 cm proximal to its attachment on the calcaneus, or as dysfunction of the attachment of the tendon onto the calcaneus, i.e., insertional Achilles tendinopathy, commonly with the formation of bone spurs and calcifications in tendon proper at the insertion site (4). Achilles tendinopathy can co-occur with paratendinopathy, defined as acute or chronic inflammation and/or degenerative changes of the thin layer of tissue surrounding the tendon (4). The paratenon is a densely vascularised and innervated elastic sheath that plays a vital role in developing painful midportion Achilles (para)tendinopathy (5). A dysfunction or pathology in the Achilles tendon area significantly decreases the patients’ health-related quality of life (6, 7). Achilles tendinopathy also greatly impacts the patient’s socioeconomic status; over one-third (38%) report reduced work productivity (6).

Achilles tendinopathy can be managed using conservative and surgical therapies (8, 9). Patients with tendon disrepair and degenerative tendinopathy might benefit from physical interventions that enhance cell activity, protein expression, and remodeling of the extracellular matrix (10). Such treatments include sound waves (11, 12) and shock waves (13, 14), among others. Both are mechanical waves, but their physical characteristics differ (15, 16). The findings of studies evaluating the effects of shock wave therapy in patients with Achilles tendinopathy are inconclusive (17, 18). The therapeutic efficacy of mechanotherapy for Achilles tendinopathy has so far been mainly measured with scales and questionnaires whereby patients subjectively assess their complaints (11, 13, 14, 19, 20). Objective measurements are not commonly used (21–24).

Degenerative lesions in the Achilles tendon reduce tendon stiffness and cause tensile mechanical changes (25, 26). Pain and swelling impair the function of the muscle–tendon unit (27). Reduction of the ankle plantar flexion and dorsiflexion moments during the mid-stance and terminal swing phases (28) may lead to difficulty with normal gait and push-off. We, therefore, formed a research hypothesis that pain relief and reduced edema and stiffness associated with prolonged rest in the Achilles tendon seen after radial shock wave/ultrasound therapy would change the dynamics of step initiation. It was also hypothesised that the magnitude of such change would be related to therapy type and measurement conditions.

The primary aim of the present study was to investigate and compare the therapeutic efficacy of two different mechanotherapy types (radial shock wave and ultrasound therapies) for non-insertional Achilles tendinopathy. Another aim was to determine whether force platform posturography might play a role as an adjunct to the objective assessment of patients with degenerative conditions of the Achilles tendon.

## Materials and methods

Based on the Consolidated Standards of Reporting Trials (CONSORT) guidelines (29), this was a 5-year (October 2017–May 2022) prospective, randomised, controlled clinical trial. The project was approved by the local research ethics committee (approval number: 5/2016) and registered in the Australian and New Zealand Clinical Trials Registry (no. ACTRN12617000860369; date registered: 9.06.2017).

The entire research project was divided into several parts. We recently published the results of the first part, which focused on the analysis of pain intensity (according to the Visual Analogue Scale) and postural sway during quiet standing and stepping tasks after mechanotherapy treatments (23, 24). This study presents the scores of the Victorian Institute of Sport Assessment–Achilles (VISA-A) questionnaire and posturographic measurements of step initiation performed under two different conditions (non-perturbed and perturbed transit).

### Patients

The trial screening procedure was applied to patients of orthopedic and physiotherapy outpatient units, whose eligibility to participate was assessed by an orthopedic surgeon based on medical history and physical examination (including ultrasound). The following inclusion criteria were used: pain over the main body of the Achilles tendon 2 to 6 cm proximal to its insertion lasting longer than 3 months, midportion tendon abnormalities identified on ultrasound, and participation in recreational activities (23). Individuals were considered recreationally active if they participated in aerobic activity for 80 min at moderate intensity less than or equal to twice a week (30). Ultrasound images showed hypoechoic thickening of the midportion of the Achilles tendon with focal disruption of its collagen fibres. Colour-coded Doppler ultrasound revealed an increase in tendon vascularity. The exclusion criteria have been precisely described in our recent study (23) and included patients under 18 years of age, pregnancy, thrombophlebitis, atherosclerosis, renal failure, local lower limb infection, neoplastic disease, history of Achilles tendon surgery, knee instability, ankle instability, lower extremity length discrepancy, anticoagulant therapy, physical therapy during 6 weeks preceding the study, corticosteroid injections during 6 weeks preceding the study, type 1 diabetes mellitus and type 2 diabetes mellitus with insulin therapy, rheumatoid arthritis and other rheumatoid diseases, cardiac pacemaker, cardiac arrhythmia, cardiovascular insufficiency, patellar tendinitis, bilateral Achilles tendinopathy, history of stroke (ischemic, hemorrhagic), neoplasms, cardiac pacemaker, pathological skin conditions, coagulation disorders, arteriosclerosis, and thromboangiitis obliterans.

**Figure 1:**
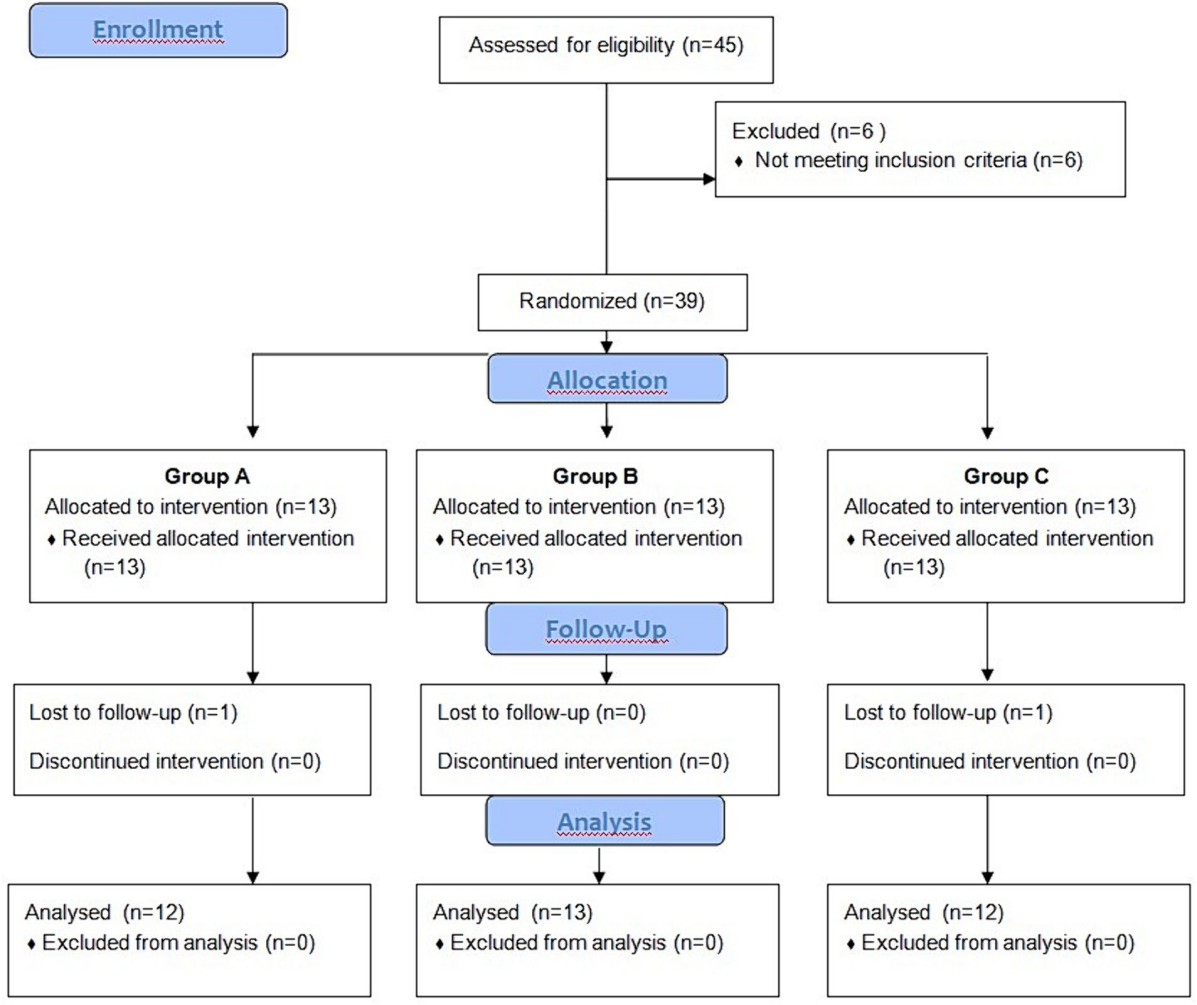
Flowchart of the trial from the baseline.

Forty-five patients with non-insertional Achilles tendinopathy were selected. An orthopedic surgeon, who had been blinded regarding the type of therapeutic intervention, checked all inclusion criteria. They enrolled 39 patients and excluded six (Figure 1).

Three groups were formed of patients who consented to participate in the study: radial shock wave therapy (RSWT) (group A), ultrasound therapy (group B), and placebo ultrasound (group C). Randomization involved the use of sequentially numbered envelopes.

Table 1 shows the baseline characteristics of the patients in all groups (23).

**Table 1:** Characteristics of the study participants.

Characteristics	Group A (*n* = 13) mean ± SD or *n* (%)	Group B (*n* = 13) mean ± SD or *n* (%)	Group C (*n* = 13) mean ± SD or *n* (%)	*p*-value
Age (years)	42 ± 11.42	36.69 ± 11.57	34 ± 11.32	*p* > 0.05*
Sex				
Female	2 (15.4%)***	4 (30.8%)	8 (61.5%)***	*p* = 0.04**
Male	11 (84.6%)	9 (69.2%)	5 (38.5%)	
Body weight (kg)	80.46 ± 10.95	79.46 ± 9.25	73.53 ± 16.08	*p* > 0.05*
Height (m)	1.79 ± 0.09	1.79 ± 0.08	1.77 ± 0.12	*p* > 0.05*
Affected limb				*p* > 0.05**
Left	6 (46.2%)	4 (30.8%)	4 (30.8%)	
Right	7 (53.8%)	9 (69.2%)	9 (69.2%)	
Duration of symptoms (mo)	8.84 ± 8.68	9.07 ± 7.64	7.53 ± 5.07	*p* > 0.05*

*Kruskal*–*Wallis test; **Chi-square test; ***Fisher’s exact test: *p* = 0.02. Bold values indicate statistically significant differences (*p* < 0.05).

The patients from groups B and C were blinded to the type of treatment. However, the treatment-providing physiotherapist, principal investigator, and radial shock wave therapy receivers were aware of the therapy type. The trial was conducted in the physiotherapy outpatient unit and the Human Motor Behavior laboratory of the Academy of Physical Education in Katowice, Poland.

### Therapy

A radial shock wave device (ShockMaster 500, Gymna-Uniphy) with a standard 15-mm pneumatic applicator was used. Each RSWT session comprised two phases. During the first phase, shocks were delivered to the Achilles tendon (2000 shocks/10 Hz/3 bars), and during the second phase, they were delivered to the gastrocnemius muscle (2000 shocks/10 Hz/3 bars). Each patient in group A received three treatments at 7-day intervals.

Ultrasonic waves were applied to the Achilles tendon in circular movements with a 4-cm^2^ transducer head (Pulson 400, Gymna). Pulsed ultrasonic waves (with 50% duty cycle) of 3 MHz frequency and 1.0 W/cm^2^ power density were used. The length of the treatment session was proportional to the size of the treatment area—each square centimeter was treated with ultrasound energy for 2 min. Each patient received the treatment once daily, i.e., 10 treatments during 2 weeks.

A Pulson 400 (Gymna) was also used for the placebo ultrasound therapy. The ultrasound machine’s parameters and treatment procedures were identical to those of group B, except that sound waves were not generated.

During the first 2 weeks of treatment, deep friction massage (six sessions) was applied to members of all experimental groups (23, 24). It would have been unethical to leave patients in the placebo group without any form of therapy for the entire research project. Hence, we applied deep transverse massage to all research groups for the first 2 weeks of the experiment.

### Outcome assessment

Before the intervention and at weeks 1 and 6 after the treatment, the patients were evaluated using the Victorian Institute of Sport Assessment–Achilles (VISA-A) questionnaire and posturographic measurements of step initiation performed on the force platforms under two different conditions (non-perturbed and perturbed transit).

All study participants provided subjective assessment (VISA-A). However, for personal reasons, two patients refused to participate in the follow-up posturographic measurements at 6 weeks post-therapy.

The VISA-A questionnaire consists of eight questions regarding pain (questions 1–3), function (questions 4–6), and activity (questions 7 and 8). The maximum score for questions one through seven is 10; question 8 yields a maximum of 30 points. The total score of 100 points indicates no abnormalities within the tendon, while 0 represents the most severe condition (31).

The posturographic measurement station for step initiation tasks consisted of two (A and B) force platforms (AMTI, AccuGait, Watertown, MA, United States), a charge amplifier, and a computer. Digital output from the platform was recorded at 100 Hz using AMTI’s NetForce software. The offline raw data were filtered using a Butterworth low pass filter with a cutoff frequency of 6 Hz (MATLAB, MathWorks, Natick, MA, United States) (32).

**Figure 2:**
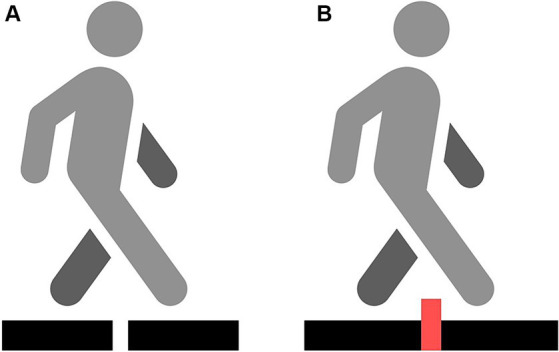
Two different conditions: non-perturbed transition **(A)** and obstacle crossing transition **(B)**.

Assessment of step initiation consisted of two trials, namely, non-perturbed and perturbed transit (with an obstacle inserted between two platforms) (Figure 2). The step initiation procedure was conducted as described in a study by Stania et al. (33).

Statistical analyses used the means of the study variables from three-step initiation repetitions within each trial. Platform change started each time at a sound signal. The procedure of step initiation was performed with the affected and unaffected limbs in random order.

The recording of the centre of foot pressure (COP) displacements was divided into three phases: phase 1—quiet standing before step initiation, phase 2—transit, and phase 3—quiet standing until measurement completion. The recording was divided into phases using an algorithm that had already been presented by Stania et al. (33). The following variables of COP displacement were determined for phases 1 and 3: sway range (raCOP) [cm] and mean velocity of COP (vCOP) [cm/s] in the sagittal (_AP_) and frontal (_ML_) planes. The following variables were calculated for phase 2: transit time–time from exit from stability until gaining post-transit stability [s]; and double-support period—time when one foot was in contact with platform A and the other with platform B [s].

### Statistical analysis

The Shapiro–Wilk test was used to check the data for normal distribution, while variance homogeneity was investigated using Levene’s test. The homogeneity of the patients’ age, body weight, height, intensity of rest pain, and tendinopathy duration distribution was analyzed with the Kruskal–Wallis test by ranks. The distribution of the remaining variables, i.e., sex and tendinopathy location (right vs. left limb), was tested using the Chi-square test of independence.

Percentage change in the VISA-A score was derived from the following formula:

$$X\left[\%\right]=\frac{\left(X_{a}-X_{b}\right)}{X_{b}}\;x\;100\left[\%\right]$$

where: X – percentage change in the VISA-A score; X_b_ – mean VISA-A score: (1) before treatment, (2) at 1-week post-treatment; X_a_ – mean VISA-A score: (1) at 1-week post-treatment, (2) at 6-week post-treatment.

The three-way repeated measures ANOVA with a 3 × 2 × 2 factorial design (timepoint × limb condition × platform arrangements) was used to analyze the posturographic and temporal parameters of step initiation phases. All two- and three-way interactions were analyzed. The ANOVA results were used to calculate the F-statistics for each main effect and interaction. The post-hoc comparisons were performed using the *Bonferroni* test. Mauchly’s sphericity test was used to validate a repeated measures ANOVA. The Greenhouse–Geisser correction was applied to adjust for a lack of sphericity in a repeated measures ANOVA. In all tests, the level of statistical significance was set at *p* ≤ 0.05.

## Results

### Subjective assessment

At 6 weeks after therapy, all groups exhibited significantly increased VISA-A scores against the measurement at week 1 after therapy (*p* = 0.01). In Group A, the mean VISA-A score at 6 weeks of therapy completion was significantly higher than the pre-intervention score (*p* = 0.04) (Table 2). The percentage change in VISA-A scores obtained at 6 weeks of therapy completion compared to pre-therapy scores, as well as in VISA-A scores obtained at 6 weeks of therapy completion compared to post-therapy scores at 1 week, was significantly greater in group A than in group B (Table 3).

**Table 2:** Changes in the mean VISA-A function scores (±SD) in groups A, B, and C.

Group	Measures of spread	Before therapy (T_0_)	1 week after therapy (T_1_)	6 week after therapy (T_2_)	p*	p** T_0_ – T_1_	p** T_1_ – T_2_	p** T_0_ – T_2_
A	X ± SD	65.54 ± 20.06	72.08 ± 19.09	87.76 ± 8.77	***p* < 0.0001**	*p* > 0.05	**0.01**	**0.04**
	Me	73	76	90				
	(Q1; Q3)	(50; 78)	(60; 85)	(83; 95)				
B	X ± SD	74.23 ± 12.98	81.08 ± 14.67	83.31 ± 15.09	***p* < 0.001**	*p* > 0.05	**0.01**	*p* > 0.05
	Me	74	85	90				
	(Q1; Q3)	(68; 84)	(71; 93)	(71; 97)				
C	X ± SD	72.85 ± 12.37	73.85 ± 11.88	75.69 ± 12.79	***p* < 0.01**	*p* > 0.05	**0.01**	*p* > 0.05
	Me	77	78	78				
	(Q1; Q3)	(62; 82)	(66; 82)	(66; 86)				

Me – median; Q1 – lower quartile; Q3 – upper quartile.

p* – Kruskal–Wallis ANOVA by ranks.

p** – the post-hoc test for Kruskal–Wallis ANOVA by ranks. Bold values indicate statistically significant differences (*p* < 0.05).

**Table 3:** Percentage change for VISA-A function scores (±SD) in groups A, B, and C.

Inter-assessment percentage change	Group A		Group B		Group C		p*	Inter-group comparisons: A-B and B-C (*p* post-hoc**)
	X [%]	SD	X [%]	SD	X[%]	SD		
T_0_ – T_1_	18.77	25.85	9.31	7.64	1.55	2.22	**0.002**	A-B: *p* > 0.05 B-C: **0.04**
T_1_ – T_2_	23.75	36.48	2.86	4.93	2.48	5.34	**0.001**	A-B: **0.01** B-C: *p* > 0.05
T_0_ – T_2_	46.37	48.87	12.59	11.38	4.06	5.58	**0.002**	A-B: **0.01** B-C: *p* > 0.05

T_0_ – before therapy; T_1_ – 1 week after therapy; T_2_ – 6 weeks after therapy.

p* – Kruskal–Wallis ANOVA by ranks.

p** – the post-hoc test for Kruskal–Wallis ANOVA by ranks. Bold values indicate statistically significant differences (*p* < 0.05).

### Objective assessment

Statistically significant changes were only noted between some posturographic parameters of step initiation tasks.

### Phase 1

The four-way interaction effect of group × timepoint × limb condition × platform arrangements was significant for raCOP in the sagittal plane (*F*(4,68) = 3.35; *p* = 0.01). However, the *Bonferroni* post-hoc test did not reveal any statistically significant differences in the values of the raCOP_AP_ (*p* > 0.05).

The three-way ANOVA demonstrated the treatment type that affected most of the measured variables: raCOP_ML_ (*F*(2,68) = 4.42; *p* = 0.02), vCOP_AP_ (F(2,68) = 4.59; *p* = 0.02) and vCOP_ML_ (F(2,68) = 3.91; *p* = 0.003). The *Bonferroni* post-hoc test showed that the means of all those variables were significantly smaller for group A than for group B patients (*p* < 0.05).

Platform arrangements also had an effect on the raCOP_ML_ (*F*(1,68) = 11.59; *p* = 0.002) and vCOP_AP_ (F(1,68) = 14.43; *p* < 0.001). The post-hoc test revealed that the mean values of the variables were significantly greater for perturbed step initiation compared to non-perturbed trial (*p* < 0.05).

### Phase 2

**Figure 3:**
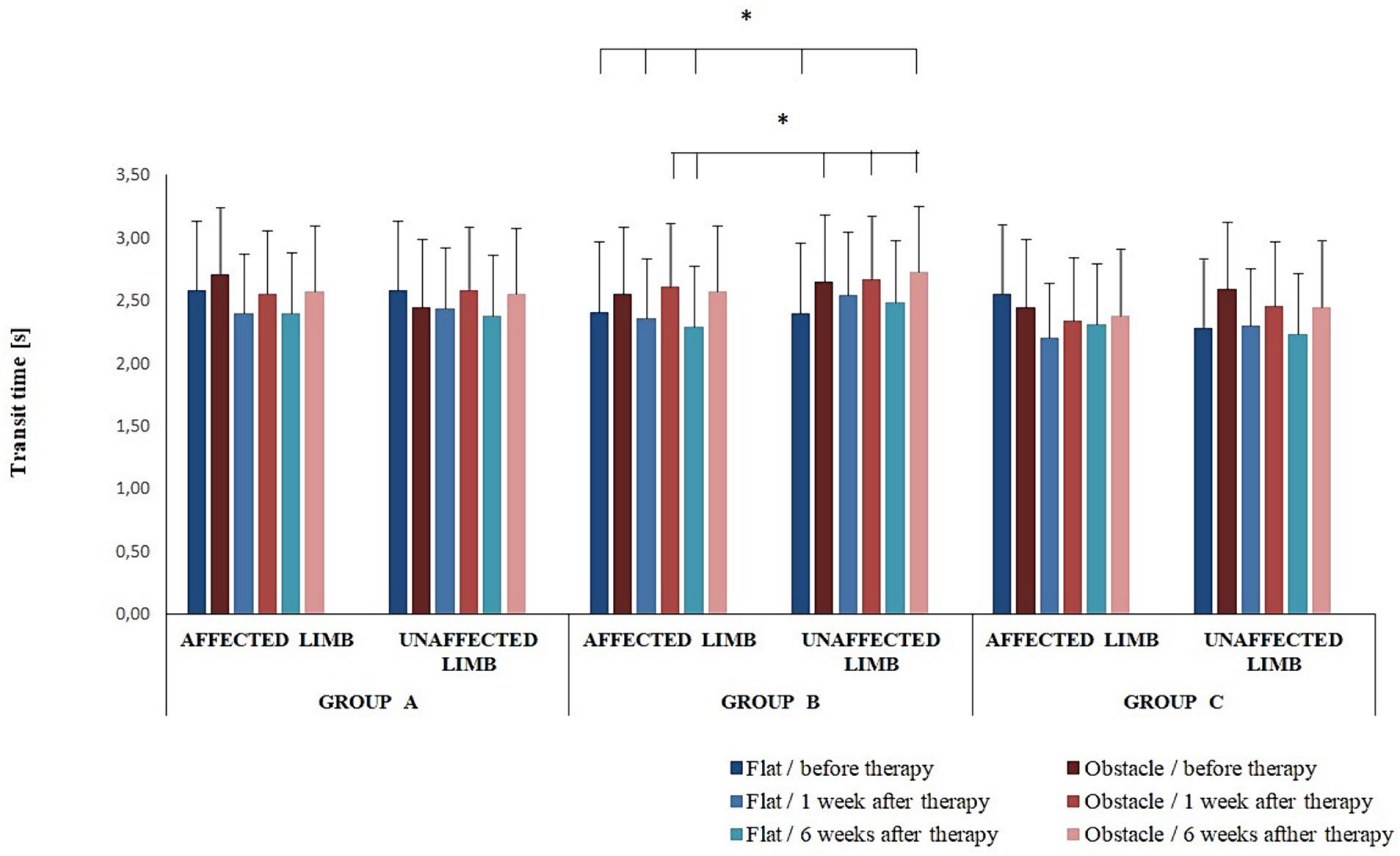
Changes in the mean transit time (± SD) in all experimental groups during the entire observation period. Horizontal lines with vertical dashes indicate statistically significant differences (*p* < 0.05).

The four-way interaction effect of group × timepoint × limb condition × platform arrangements was significant for transit time (*F*(4,68) = 2.65; *p* = 0.04) (Figure 3). The *Bonferroni* post-hoc test revealed statistically significant differences in transit time between group B participants (Figure 3).

**Figure 4:**
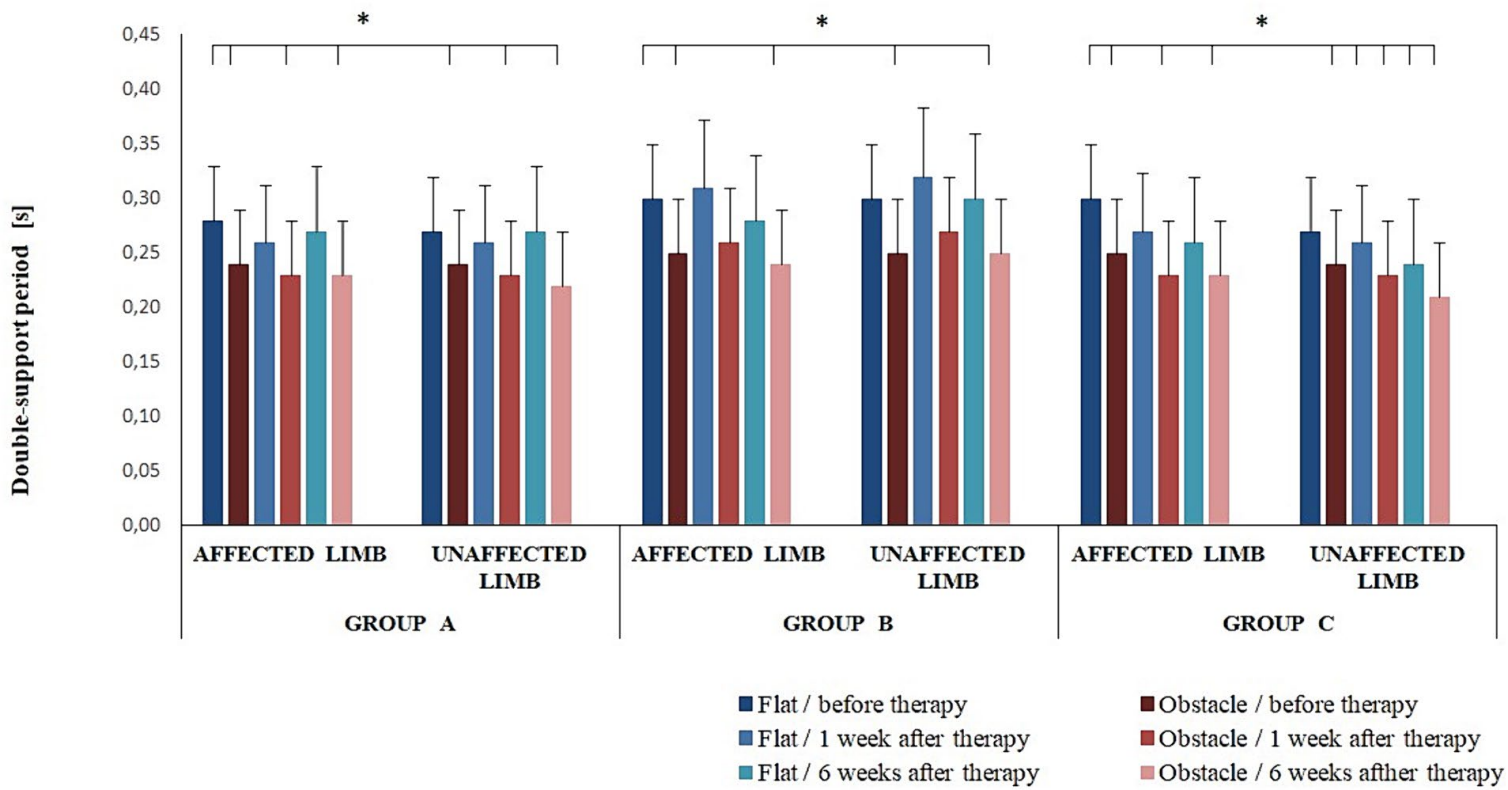
Changes in the mean double-support period (± SD) in all experimental groups during the entire observation period. Horizontal lines with vertical dashes indicate statistically significant differences (*p* < 0.05).

The post-hoc test also showed the duration of double-support was significantly longer for pre-intervention non-perturbed transit initiated with the affected limb compared to perturbed transit at 1 and 6 weeks after therapy (*p* < 0.05) (Figure 4). In addition, in all experimental groups, the duration of double-support was significantly longer for pre-intervention non-perturbed transit initiated with the affected limb compared to perturbed transit initiated with the unaffected limb at 1 and 6 weeks of therapy completion (*p* < 0.05) (Figure 4).

The three-way ANOVA revealed an effect of the platform arrangement on transit time (*F*(1,68) = 37.22; *p* < 0.001) and double-support period (F(1,68) = 61.05; *p* < 0.001). The *Bonferroni* post-hoc test showed transit time was significantly longer for the perturbed vs. non-perturbed trial (*p* < 0.05); a reverse relationship was observed for the double-support period (*p* < 0.05).

### Phase 3

The four-way interaction effect of group × timepoint × limb condition × platform arrangements was not significant for any of the measured posturographic variables (*p* > 0.05).

The three-way ANOVA revealed an effect of the platform condition on raCOP_ML_ (F(1,68) = 18.68; *p* < 0.001), vCOP_AP_ (F(1,68) = 25.36; *p* < 0.001), and vCOP_ML_ (F(1,68) = 15.64; *p* < 0.001). The means of all those variables were significantly greater for perturbed step initiation compared to non-perturbed trial (*p* < 0.05).

## Discussion

The post-therapy subjective assessment of tendinopathy-related complaints with the VISA-A questionnaire revealed significant improvement in all experimental groups; the therapy was shown to be the most effective in group A (Tables 2, 3). However, a majority of changes captured by successive measurements on force platforms were too small and inconsistent to make the analysis of posturographic parameters of quiet standing before and after step initiation useful for the evaluation of treatment efficacy in patients with non-insertional Achilles tendinopathy. Since the three-way ANOVA did not reveal any effect of limb condition on the variables under consideration, Achilles tendinopathy does not seem to reduce posturographic locomotor performance on simple motor tasks.

An analysis of VISA-A scores showed an advantage of RSWT over ultrasounds (Table 3), whereas a meta-analysis by Punnoose et al. (34) did not demonstrate statistically significant differences between extracorporeal shock wave therapy (ESWT) and other interventions on the pooled VISA-A scores (95% CI: −15.02, 26.51; *p* = 0.59). These differences might possibly result from different baseline characteristics of patients with Achilles tendinopathy. In our experiment, the mean pre-intervention VISA-A score was over 65, and the mean duration of symptoms was approximately 8 months. In the primary research by Rompe et al. (35, 36), included in Punnoose et al.’s analysis (34), the mean pre-intervention VISA-A score was markedly lower (below 53.5 points), and symptom duration was longer and more variable (9.2–26.3 months). According to the continuum model of tendon pathology proposed by Cook and Purdam (10, 37), the early stage of tendon dysfunction, i.e., reactive tendinopathy, is a reversible condition. This might account for significant post-therapy function improvement in our study, as revealed by the subjective assessment with the VISA-A questionnaire.

Step initiation is a transition phase between quiet standing and the gait cycle. It is a complex motor task that requires the integration of sensory information arising from the proprioceptive, vestibular, and visual systems (38). Gait initiation also depends on skeletal muscle activities, ground reaction forces, movements of the centre of pressure and centre of mass as well as joint motions (39), and anticipatory postural adjustments (40). Lifting the swing foot off the ground to make a step, i.e., moving from a bipedal to an unipedal stance, reduces the mediolateral base of support. If the centre of mass is not moved above the limits of the resultant base of support (the stance foot), the body will start bending excessively toward the swing leg side, resulting in a risk of falling (40). Such instability is prevented by anticipatory postural adjustments that precede postural perturbations associated with voluntary movements (40).

Gait disturbances seen in individuals with Achilles tendinopathy include changes in spatial–temporal gait parameters, i.e., reduced step length and walking speed and an increase in double-limb support (28). The pre-intervention double-limb support time was also longer in our experiment. Post-therapy assessment (groups A, B, and C) revealed statistically significant intragroup changes in double-support throughout the observation period, while intergroup differences did not reach the level of statistical significance.

However, a three-way ANOVA revealed an effect of therapy type on raCOP_ML_, vCOP_AP_, and vCOP_ML_ for quiet standing before step initiation; vCOP_AP_ for quiet standing after transit phase was also affected. Overall, postural control in quiet standing was more efficient in patients who received RSWT than in the ultrasound group, thus implying that a relationship existed between shock wave therapy and smaller COP displacement, similar to our recent study (23).

As expected, non-perturbed transit between platforms was an easier motor task for all study participants. The presence of an obstacle challenges postural stability during gait initiation. However, the central nervous system is capable of predicting obstacle-related balance problems and modifies the spatiotemporal parameters of anticipatory postural adjustments accordingly (40).

The clinical improvement observed in all experimental groups (Table 2) might have been—to a greater or lesser degree—a result of deep friction massage applied in all participants. Manual techniques may have mechanical effects on microstructure abnormalities in affected tendons (41). Deep friction massage is essential for releasing fibrotic adhesions between the Achilles tendon and the surrounding paratenon (especially in chronic midportion Achilles tendinopathy), as it promotes the restoration of tendon gliding.

The most common clinical sign found on examination of non-insertional Achilles tendinopathy is a spindle-shaped thickening, usually located 2 to 6 cm proximal to the tendon’s calcaneal insertion. A squeeze test that involves pinching the thickening with two fingers often reproduces the pain. The stiffness of the gastrocnemius-soleus complex on passive stretching is another common complaint, especially in athletes with a chronic condition (42). Based on histopathological studies, pathologies of the Achilles tendon include midportion tendinosis, which can be coupled with small-size focal tears of the collagen fibres, paratendinitis, and paratendinopathy with or without pathological changes of the tendon tissue (43). These lesions should be considered separate disease entities, although they often coexist and share clinical features. Diagnostic tests should facilitate differentiation between the different pathologies of the Achilles tendon, retrocalcaneal bursitis, and superficial calcaneal bursitis (4); however, the criteria for qualifying patients for experiments evaluating the efficacy of shock wave and ultrasound therapy focused primarily on the location of tendinopathy within the Achilles tendon, without reference to potential pathological changes of the paratenon (21, 22, 34, 44). Hence, there is a need for randomised clinical trials verifying the effectiveness of mechanotherapy depending on the phenotypes of Achilles pathology.

The molecular mechanisms of shock waves and ultrasound action on tendon tissue are quite complex (45, 46). It is believed that the regenerative effects of ESWT are derived from mechanotransduction, where the mechanical energy is converted to biochemical energy in the tendon cell (46). Shock waves promote tenocyte viability and proliferation as well as the expression of typical tendon markers and anti-inflammatory cytokines (47, 48). ESWT may initiate tendon tissue regeneration by promoting pro-inflammatory (interleukin-6) and catabolic processes (interleukin-8) to remove damaged matrix constituents (49). It also facilitates collagen synthesis by upregulating proliferating cell nuclear antigen and *transforming growth factor* ß *1* (50). *In vitro* study (51) demonstrated that therapeutic ultrasound also increased fibroblast proliferation, enhanced the synthesis of collagen, non-collagenous proteins, and production of interleukin-8, vascular endothelial growth factor, and basic fibroblast growth factor. Ultrasounds mainly stimulate types I and III collagen expression (52), the predominant proteins in damaged regions of the tendon. Ultrasonic waves also stimulate tendon cell migration and proliferation (53), which is crucial for tendon healing (45). The mechanical impact of ultrasound is smaller compared to shock waves (15, 16), which might account for a greater increase in VISA-A scores observed in group A compared to groups B and C (Table 3).

Shock wave treatments for patients with tendinopathy do not cause a rapid and significant change; instead, they contribute to the gradual healing of the affected tissues (54). The main limitation of the study was the lack of long-term follow-up. A posturographic examination at a timepoint distant from mechanotherapy completion might have been sensitive enough to detect changes in postural control in patients with non-insertional Achilles tendinopathy who had undergone the treatment. When designing the experiment, we did plan a follow-up assessment at 24 weeks of therapy completion. Regrettably, approximately 40% of the patients refused to participate or failed to arrive for personal or health-related reasons.

Another limitation was that a small number of patients were in the experimental groups. Increasing the sample size may have yielded significant differences between the variables studied. In addition, in the present experiment, postural control of patients with Achilles tendinopathy was primarily assessed during quiet standing before and after step initiation rather than during step initiation. Regarding step initiation, it is essential to explore anticipatory postural adjustments (APAs) from COP data and their preparatory (imbalance), unloading, and transitory phases (55). We did not analyse the temporal and spatial parameters of APAs from COP, which would allow us to assess postural control during transient locomotor tasks in patients with non-insertional Achilles tendinopathy. Such an analysis could provide more information regarding the efficacy of mechanotherapy.

The results of the RSWT group were compared to those obtained in the group treated with ultrasounds. The latter were compared with the placebo group. We are, of course, aware that, under the assumptions of evidence-based medicine, randomised *placebo-*controlled trials are the *gold standard* research design for treatment efficacy evaluation. However, the majority of individuals are aware that shock wave therapy could be associated with painful sensations, and we believed the absence of any discomfort could make the participants suspicious (23). Therefore, we did not create a placebo control for RSWT; we consider this the fourth limitation. However, there was a placebo group for ultrasound therapy as patients did not find it uncomfortable.

## Conclusion

A follow-up subjective assessment (VISA-A) performed at 6 weeks of treatment completion showed that RSWT was significantly more effective than ultrasound therapy for alleviation of pain intensity as well as improvement of function and activity in patients with non-insertional Achilles tendinopathy.

Objective data registered by force platforms during quiet standing before and after the step initiation task did not prove useful for tracing the progress of patients with non-insertional Achilles tendinopathy between consecutive therapy interventions.

## Data availability statement

The original contributions presented in the study are included in the article/supplementary material, further inquiries can be directed to the corresponding author.

## Ethics statement

The studies involving humans were approved by The Research Ethics Committee from The Academy of Physical Education in Katowice, Poland. The studies were conducted in accordance with the local legislation and institutional requirements. The participants provided their written informed consent to participate in this study.

## Author contributions

MS: Conceptualization, Formal analysis, Investigation, Methodology, Supervision, Writing – original draft. KS: Methodology, Software, Writing – review & editing. GJ: Methodology, Software, Writing – review & editing. TK: Writing – review & editing, Formal analysis. PK: Conceptualization, Investigation, Methodology, Supervision, Writing – review & editing.

## References

[1] Järvinen T Kannus P Maffulli N Khan K. Achilles tendon disorders: etiology and epidemiology. Foot Ankle Clin. (2005) 10:255–66. doi: 10.1016/j.fcl.2005.01.013 15922917

[2] Singh A Calafi A Diefenbach C Kreulen C Giza E. Noninsertional tendinopathy of the Achilles. Foot Ankle Clin. (2017) 22:745–60. doi: 10.1016/j.fcl.2017.07.006 29078826

[3] Chimenti RL Cychosz CC Hall MM Phisitkul P. Current concepts review update: insertional Achilles tendinopathy. Foot Ankle Int. (2017) 38:1160–9. doi: 10.1177/1071100717723127, PMID: 28789557 PMC5956523

[4] van Dijk C van Sterkenburg M Wiegerinck J Karlsson J Maffulli N. Terminology for Achilles tendon related disorders. Knee Surg Sport Traumatol Arthrosc. (2011) 19:835–41. doi: 10.1007/s00167-010-1374-z, PMID: 21222102 PMC3076576

[5] Ricci V Mezian K Chang KV Tamborrini G Jačisko J Naňka O. Ultrasound-guided injection of the achilles paratenon: a cadaveric investigation. Foot Ankle Surg. (2024) 30:313–8. doi: 10.1016/j.fas.2024.01.005 38296758

[6] Sleeswijk Visser TSO Van Der Vlist AC Van Oosterom RF Van Veldhoven P Verhaar JAN De Vos RJ. Impact of chronic Achilles tendinopathy on health-related quality of life, work performance, healthcare utilisation and costs. BMJ Open Sport Exerc Med. (2021) 7:e001023. doi: 10.1136/bmjsem-2020-001023, PMID: 33868707 PMC8006822

[7] Lewis TL Yip GCK Robertson K Groom WD Francis R Singh S. Health-related quality of life in patients with Achilles tendinopathy: comparison to the general population of the United Kingdom. Foot Ankle Surg. (2022) 28:1064–8. doi: 10.1016/j.fas.2022.02.018, PMID: 35279393

[8] Tarantino D Mottola R Resta G Gnasso R Palermi S Corrado B. Achilles tendinopathy pathogenesis and management: a narrative review. Int J Environ Res Public Health. (2023) 20:1–18. doi: 10.3390/ijerph20176681, PMID: 37681821 PMC10487940

[9] Tarantino D Palerm S Sirico F Balato G D’addona A Corrado B. Achilles tendon pathologies: how to choose the best treatment. J Hum Sport Exerc. (2020) 15:1300–21. doi: 10.14198/jhse.2020.15.Proc4.29

[10] Cook JL Purdam CR. Is tendon pathology a continuum? A pathology model to explain the clinical presentation of load-induced tendinopathy. Br J Sports Med. (2009) 43:409–16. doi: 10.1136/bjsm.2008.051193, PMID: 18812414

[11] Chester R Costa ML Shepstone L Cooper A Donell ST. Eccentric calf muscle training compared with therapeutic ultrasound for chronic Achilles tendon pain-a pilot study. Man Ther. (2008) 13:484–91. doi: 10.1016/j.math.2007.05.014, PMID: 17662639

[12] Hsu AR Holmes GB. Preliminary treatment of Achilles tendinopathy using low-intensity pulsed ultrasound. Foot Ankle Spec. (2016) 9:52–7. doi: 10.1177/1938640015599038, PMID: 26253528

[13] Pinitkwamdee S Laohajaroensombat S Orapin J Woratanarat P. Effectiveness of extracorporeal shockwave therapy in the treatment of chronic insertional Achilles tendinopathy. Foot Ankle Int. (2020) 41:403–10. doi: 10.1177/1071100719898461, PMID: 31924120

[14] Yan B Wan Y Zhang H Pan M Zhou C. Extracorporeal shockwave therapy for patients with chronic Achilles tendinopathy in long or short course. Biomed Res Int. (2020) 2020:1–7. doi: 10.1155/2020/7525096 PMC7441454 32851086

[15] Baker KG Robertson VJ Duck FA. A review of therapeutic ultrasound: biophysical effects. Phys Ther. (2001) 81:1351–8. doi: 10.1093/ptj/81.7.1351 11444998

[16] Ogden JA Tóth-Kischkat A Schultheiss R. Principles of shock wave therapy. Clin Orthop Relat Res. (2001) 387:8–17. doi: 10.1097/00003086-200106000-00003 11400898

[17] Charles R Fang L Zhu R Wang J. The effectiveness of shockwave therapy on patellar tendinopathy, Achilles tendinopathy, and plantar fasciitis: a systematic review and meta-analysis. Front Immunol. (2023) 14:1193835. doi: 10.3389/fimmu.2023.1193835 37662911 PMC10468604

[18] Stania M Malá J Chmielewska D. The efficacy of extracorporeal shock wave therapy as a monotherapy for Achilles tendinopathy: a systematic review and meta-analysis. J Chiropr Med. (2023) 22:294–301. doi: 10.1016/j.jcm.2023.04.003, PMID: 38205224 PMC10774612

[19] Dedes V Tzirogiannis K Polikandrioti M Dede A Mitseas A Panoutsopoulos G. Comparison of radial extracorporeal shockwave therapy with ultrasound therapy in patients with lateral epicondylitis. J Med Ultrason. (2020) 47:319–25. doi: 10.1007/s10396-019-01002-9, PMID: 31912320

[20] Abdelkader NA Helmy MNK Fayaz NA Saweeres ESB. Short- and intermediate-term results of extracorporeal shockwave therapy for noninsertional Achilles tendinopathy. Foot Ankle Int. (2021) 42:788–97. doi: 10.1177/1071100720982613, PMID: 33451253

[21] Zhang S Li H Yao W Hua Y Li Y. Therapeutic response of extracorporeal shock wave therapy for insertional Achilles tendinopathy between sports-active and nonsports-active patients with 5-year follow-up. Orthop J Sport Med. (2020) 8:232596711989811. doi: 10.1177/2325967119898118, PMID: 32030348 PMC6977229

[22] Gatz M Schweda S Betsch M Dirrichs T de la Fuente M Reinhardt N. Line- and point-focused extracorporeal shock wave therapy for Achilles tendinopathy: a placebo-controlled RCT study. Sports Health. (2021) 13:511–8. doi: 10.1177/1941738121991791, PMID: 33586526 PMC8404720

[23] Stania M Juras G Marszałek W Król P. Analysis of pain intensity and postural control for assessing the efficacy of shock wave therapy and sonotherapy in Achilles tendinopathy – a randomized controlled trial. Clin Biomech. (2022) 101:105830. doi: 10.1016/j.clinbiomech.2022.105830 36469960

[24] Stania M Pawłowski M Marszałek W Juras G Słomka KJ Król P. A preliminary investigation into the impact of shock wave therapy and sonotherapy on postural control of stepping tasks in patients with Achilles tendinopathy. Front Neurol. (2023) 14:1–15. doi: 10.3389/fneur.2023.1157335 PMC10272772 37332988

[25] Bah I Fernandes N Chimenti R Ketz J Flemister A Buckley M. Tensile mechanical changes in the Achilles tendon due to insertional Achilles tendinopathy. J Mech Behav Biomed Mater. (2020) 112:104031. doi: 10.1016/j.jmbbm.2020.104031, PMID: 32882677 PMC8056289

[26] Finnamore E Waugh C Solomons L Ryan M West C Scott A. Transverse tendon stiffness is reduced in people with Achilles tendinopathy: a cross-sectional study. PLoS One. (2019) 14:e0211863. doi: 10.1371/journal.pone.0211863, PMID: 30785895 PMC6382130

[27] Silbernagel KG Gustavsson A Thomeé R Karlsson J. Evaluation of lower leg function in patients with Achilles tendinopathy. Knee Surg Sport Traumatol Arthrosc. (2006) 14:1207–17. doi: 10.1007/s00167-006-0150-6, PMID: 16858560

[28] Kim S Yu JH. Changes of gait parameters and lower limb dynamics in recreational runners with Achilles tendinopathy. J Sport Sci Med. (2015) 14:284–9. PMC4424456 25983576

[29] Cuschieri S. The CONSORT statement. Saudi J Anaesth. (2019) 13:S27–30. doi: 10.4103/sja.SJA_559_18, PMID: 30930716 PMC6398298

[30] Bolgar MR Baker CE Goss FL Nagle E Robertson RJ. Effect of exercise intensity on differentiated and undifferentiated ratings of perceived exertion during cycle and treadmill exercise in recreationally active and trained women. J Sports Sci Med. (2010) 9:557–63. PMID: 24149781 PMC3761819

[31] Robinson JM Cook JL Purdam C Visentini PJ Ross J Maffulli N. The VISA-A questionnaire: a valid and reliable index of the clinical severity of Achilles tendinopathy. Br J Sports Med. (2001) 35:335–41. doi: 10.1136/bjsm.35.5.335, PMID: 11579069 PMC1724384

[32] Stania M Sarat-Spek A Blacha T Kazek B Juras A Słomka K. Rambling-trembling analysis of postural control in children aged 3-6 years diagnosed with developmental delay during infancy. Gait Posture. (2020) 82:273–80. doi: 10.1016/j.gaitpost.2020.09.018, PMID: 32992099

[33] Stania M Sarat-Spek A Blacha T Kazek B Slomka KJ Emich-Widera E. Step-initiation deficits in children with faulty posture diagnosed with neurodevelopmental disorders during infancy. Front Pediatr. (2017) 5:239. doi: 10.3389/fped.2017.00239 29164088 PMC5675841

[34] Punnoose A. Extracorporeal shock wave therapy for Achilles and Patellar tendinopathy: Meta-analysis and a systematic review of the literature. J Physiother Phys Rehabil. (2017) 2:1–7. doi: 10.4172/2573-0312.1000124

[35] Rompe J Nafe B Furia JP Maffulli N. Eccentric loading, shock-wave treatment, or a wait-and-see policy for tendinopathy of the main body of tendo Achillis: a randomized controlled trial. Am J Sports Med. (2007) 35:374–83. doi: 10.1177/0363546506295940, PMID: 17244902

[36] Rompe J Furia J Maffulli N. Eccentric loading compared with shock wave treatment for chronic insertional Achilles tendinopathy: a randomized, controlled trial. J Bone Jt Surg Ser A. (2008) 90:52–61. doi: 10.2106/JBJS.F.01494, PMID: 18171957

[37] Cook JL Rio E Purdam CR Docking SI. Revisiting the continuum model of tendon pathology: what is its merit in clinical practice and research? Br J Sports Med. (2016) 50:1187–91. doi: 10.1136/bjsports-2015-095422, PMID: 27127294 PMC5118437

[38] Yiou E Caderby T Delafontaine A Fourcade P Honeine J. Balance control during gait initiation: state-of-the-art and research perspectives. World J Orthop. (2017) 8:815–28. doi: 10.5312/wjo.v8.i11.815, PMID: 29184756 PMC5696609

[39] Park S Choi H Ryu K Kim S Kim Y. Kinematics, kinetics and muscle activities of the lower extremity during the first four steps from gait initiation to the steady-state walking. J Mech Sci Technol. (2009) 23:204–11. doi: 10.1007/s12206-008-0812-z

[40] Yiou E Artico R Teyssedre C Labaune O Fourcade P. Anticipatory postural control of stability during gait initiation over obstacles of different height and distance made under reaction-time and self-initiated instructions. Front Hum Neurosci. (2016) 10:1–16. doi: 10.3389/fnhum.2016.00449, PMID: 27656138 PMC5013047

[41] Joseph MF Taft K Moskwa M Denegar CR. Deep friction massage to treat tendinopathy: a systematic review of a classic treatment in the face of a new paradigm of understanding. J Sport Rehabil. (2012) 21:343–53. doi: 10.1123/jsr.21.4.343, PMID: 22234925

[42] Cocco G Ricci V Corvino A Abate M Vaccaro A Bernabei C. Musculoskeletal disorders in padel: from biomechanics to sonography. J Ultrasound. (2024) 27:335–54. doi: 10.1007/s40477-023-00869-2, PMID: 38578364 PMC11178742

[43] Maffulli N Khan KM Puddu G. Overuse tendon conditions: time to change a confusing terminology. Arthroscopy. (1998) 14:840–3. doi: 10.1016/S0749-8063(98)70021-0, PMID: 9848596

[44] Notarnicola A Maccagnano G Tafuri S Forcignanò MI Panella A Moretti B. CHELT therapy in the treatment of chronic insertional Achilles tendinopathy. Lasers Med Sci. (2014) 29:1217–25. doi: 10.1007/s10103-013-1510-3, PMID: 24352875

[45] Tsai WC Tang SFT Liang FC. Effect of therapeutic ultrasound on tendons. Am J Phys Med Rehabil. (2011) 90:1068–73. doi: 10.1097/PHM.0b013e31821a70be 21552108

[46] d’Agostino MC Craig K Tibalt E Respizzi S. Shock wave as biological therapeutic tool: from mechanical stimulation to recovery and healing, through mechanotransduction. Int J Surg. (2015) 24:147–53. doi: 10.1016/j.ijsu.2015.11.030, PMID: 26612525

[47] Vetrano M D’Alessandro F Torrisi M Ferretti A Vulpiani M Visco V. Extracorporeal shock wave therapy promotes cell proliferation and collagen synthesis of primary cultured human tenocytes. Knee Surg Sports Traumatol Arthrosc. (2011) 19:2159–68. doi: 10.1007/s00167-011-1534-9, PMID: 21617986

[48] Chao Y Tsuang Y Sun J Chen L Chiang Y Wang C. Effects of shock waves on tenocyte proliferation and extracellular matrix metabolism. Ultrasound Med Biol. (2008) 34:841–52. doi: 10.1016/j.ultrasmedbio.2007.11.002, PMID: 18222032

[49] Waugh C Morrissey D Jones E Riley G Langberg H Screen H. In vivo biological response to extracorporeal shockwave therapy in human tendinopathy. Eur Cell Mater. (2015) 29:268–80. doi: 10.22203/eCM.v029a20, PMID: 25978115

[50] Chen Y Wang C Yang K Kuo Y Huang H Huang Y. Extracorporeal shock waves promote healing of collagenase-induced Achilles tendinitis and increase TGF-beta1 and IGF-I expression. J Orthop Res. (2004) 22:854–61. doi: 10.1016/j.orthres.2003.10.013, PMID: 15183445

[51] Doan N Reher P Meghji S Harris M. In vitro effects of therapeutic ultrasound on cell proliferation, protein synthesis, and cytokine production by human fibroblasts, osteoblasts, and monocytes. J Oral Maxillofac Surg. (1999) 57:409–19. doi: 10.1016/S0278-2391(99)90281-1 10199493

[52] Tsai WC Pang JHS Hsu CC Chu NK Lin MS Hu CF. Ultrasound stimulation of types I and III collagen expression of tendon cell and upregulation of transforming growth factor beta. J Orthop Res. (2006) 24:1310–6. doi: 10.1002/jor.20130, PMID: 16705693

[53] Tsai WC Chen JYS Pang JHS Hsu CC Lin MS Chieh LW. Therapeutic ultrasound stimulation of tendon cell migration. Connect Tissue Res. (2008) 49:367–73. doi: 10.1080/03008200802325359, PMID: 18991090

[54] Stania M Król B Franek A Błaszczak E Dolibog P Polak A. A comparative study of the efficacy of radial and focused shock wave therapy for tennis elbow depending on symptom duration. Arch Med Sci. (2020) 17:1686–95. doi: 10.5114/aoms.2019.81361, PMID: 34900050 PMC8641526

[55] Russo Y Vannozzi G. Anticipatory postural adjustments in forward and backward single stepping: task variability and effects of footwear. J Biomech. (2021) 122:110442. doi: 10.1016/j.jbiomech.2021.110442 33901937

